# Characteristics of SARS-CoV-2 Seropositivity among Emergency Department Healthcare Workers at a Tertiary Care Center in Baltimore

**DOI:** 10.3390/healthcare10030576

**Published:** 2022-03-20

**Authors:** Anna Russell, Edbert B. Hsu, Katherine Z. J. Fenstermacher, Erin P. Ricketts, Gabriella Dashler, Allison Chen, Kathryn Shaw-Saliba, Patrizio P. Caturegli, Andrew Pekosz, Richard E. Rothman

**Affiliations:** 1 Department of Emergency Medicine, School of Medicine, The Johns Hopkins University, Baltimore, MD 21205, USA; ehsu1@jhmi.edu (E.B.H.); kfenste1@jhu.edu (K.Z.J.F.); erinricketts@jhu.edu (E.P.R.); ggladfe1@jhmi.edu (G.D.); achen94@jhu.edu (A.C.); katy.saliba@nih.gov (K.S.-S.); apekosz1@jhu.edu (A.P.); rrothma1@jhmi.edu (R.E.R.); 2 Department of Pathology, School of Medicine, The Johns Hopkins University, Baltimore, MD 21205, USA; pcat@jhmi.edu; 3 Department of Molecular Microbiology and Immunology, Johns Hopkins Bloomberg School of Public Health, Baltimore, MD 21205, USA

**Keywords:** SARS-CoV-2, seroprevalence, healthcare workers, emergency department

## Abstract

Early in the COVID-19 pandemic (March–July 2020 in Baltimore), emergency department (ED) healthcare workers (HCWs) were considered to be at greater risk of contracting SARS-CoV-2. Limited data existed, however, on the prevalence of SARS-CoV-2 infection and its impact in this workforce population. We enrolled 191 ED HCWs from a tertiary academic center, administered baseline and weekly surveys, and tested them twice (July and December 2020) for serum antibodies against SARS-CoV-2 spike protein. Approximately 6% (11 of 191, 5.8%) of ED HCWs had spike antibodies in July, a prevalence that doubled by December (21 of 174, 12.1%). A positive PCR test was self-reported by 15 of 21 (71%) seropositive and 6 of 153 (4%) seronegative HCWs (*p* < 0.001). Of the total 27 HCWs who had antibodies and/or were PCR positive, none required hospitalization, 18 (67%) had a self-perceived COVID-19 illness, and 12 of the 18 reported symptoms. The median number of missed workdays was 8.5 (ranging from 2 to 21). While most seropositive ED HCWs who reported symptoms took work absences, none required hospitalization, indicating that COVID-19’s impact on staffing prior to vaccination was not as great as feared.

## 1. Introduction

Early in the pandemic, emergency department (ED) healthcare workers (HCWs) were considered among those at higher risk of contracting SARS-CoV-2, due to the high frequency and potential increased duration of contact with patients with suspected or known infections. The fast-paced, unpredictable work setting during the early phases of the pandemic was exacerbated by inconsistent availability of personal protective equipment (PPE) and variability in PPE training and compliance. The requirement for the performance of emergent, unplanned aerosol-generating procedures known to be associated with increased transmission risk also raised concerns [1,2].

During the very early phases of the pandemic, one review suggested that HCWs are at increased risk of contracting COVID-19 but that protective measures (e.g., PPE, hand-washing) significantly mitigated that risk [2]. Further concerns regarding HCWs becoming infected were raised, given risk of their spreading the virus not only to their household contacts and the communities they interface with but also to patients and other hospital personnel [1]. Finally, there was great concern that COVID-19 would place immense strain on the healthcare system and that severe illness and absenteeism among HCWs would lead to workforce shortages at hospitals [3,4]. This has become a critical issue in the massive surge of COVID-19 cases caused by the Omicron variant. While up to 5% of HCW cases resulted in severe illness, the vast majority of HCWs experienced relatively low rates of severe disease [5,6]. Despite this, interest in the evolving trends of COVID-19 infection among HCWs remains, including the degree to which illness played a role in workforce shortages during the early phases of the pandemic.

Serological studies have enabled comparative assessments of COVID-19 burden in various populations, given that they permit the detection of cases that would otherwise be missed or underreported due to lack of consistent testing and/or mild symptoms. Two systematic reviews completed in 2020, which included publications from the beginning of the pandemic through the summer of 2020, showed an average HCW SARS CoV-2 seropositivity rate of 7% and 8.7% [5,7] but with high variability between individual studies (from 0% to 45%). Several findings from these studies have been frequently reported. Rates of seropositivity were directly linked with department or job function, leading to the identification of certain groups, including nurses, with higher rates of positivity [8,9,10,11]; community exposure to COVID-19 was identified as a strong independent risk for HCW seroconversion with significantly higher rates of seropositivity among HCWs with household exposures [8,9]; notably, however, one study reported that up to one-third of COVID-19 cases in HCWs were directly linked to an occupational exposure [11]; younger HCWs tended to have higher positivity rates [8,11,12,13]; and higher seropositivity was reported among non-Hispanic, Black HCWs relative to other racial and ethnic groups [8,10,11].

To our knowledge, prior serosurvey studies focusing on HCWs (including clinical and non-clinical workers) have not reported how SARS-CoV-2 seropositivity evolved over an extended period during the early phases of the pandemic. To date, we found only two studies that describe seroconversion trends for HCWs—however, both were limited to a duration of one to two months [14,15]. In addition, most studies have not contextualized the observed seropositivity rates of HCWs with the seropositivity and/or positive test rates in the local community, despite this being a large source of infection. Our HCWs include direct frontline clinicians and non-clinician staff who work in the ED, given the importance of both workforce groups to ED functionality, and early concerns in the pandemic that both were at increased risk of contracting COVID-19 [16]. Here, we describe changes observed in SARS-CoV-2 seropositivity among ED HCWs who worked in a tertiary care center during the very early phases of the pandemic, over a six-month period. We also identify demographic and work-related characteristics and associated infection rates and report duration of work absenteeism among ED HCWs who were PCR positive and/or seropositive during the study period. We discuss our findings in the context of observed local trends in infection in Baltimore City during the same period.

## 2. Materials and Methods

This was a prospective longitudinal study of 191 ED HCWs who worked in the ED at The Johns Hopkins Hospital, a tertiary care academic medical center located in Baltimore, Maryland. The operational definition of ED HCW included clinical as well as non-clinical staff, a definition that has been used by CDC guidance on COVID-19 exposure among HCWs [17]. Non-clinical staff included staff who worked in the ED but were not directly responsible for clinical care, inclusive of security, environmental services, and administrative and registrar personnel. Blood samples for the serosurvey were collected in July and December 2020 and subsequently tested (see Section 2.3. IgG Sample Testing). Baseline and subsequent weekly structured surveys were sent electronically to all participants; those participants who had a positive serologic test and/or self-reported having had a PCR positive test during the study period were also asked to complete an online illness severity and a work absenteeism survey at the end of the study period.

### 2.1. Recruitment and Study Visits

Recruitment was conducted through posted flyers and email listservs distributed to all ED HCWs (inclusive of clinicians and all staff who worked in the ED). Interested participants were directed to an easy-access JHU-housed REDCap platform [18], which described the basic purpose and components of the study in more detail, with eligibility and exclusions. Participants aged 18 years or older who were full-time employees of the ED were considered eligible, which included attending physicians, fellows, residents, nurses, CNAs (clinical nurse associates), technicians, security, environmental services associates, and social workers/case workers; exclusion criteria were lack of routine access to the Internet (required to complete the survey) and inability to comply with in-person follow-up visits. Interested participants were directed to make an appointment with a study staff member, who completed the in-person informed consent in the ED. Following consent, participants had an initial blood sample collected for serologic testing and were provided instructions on completing the initial online survey. Procedures were also reviewed for completing the weekly online survey (all participants). Consenting subjects were then contacted after 5 months to schedule their 6-month follow-up blood draw visit (in December 2020). Participants who tested seropositive either at baseline or follow-up were contacted and informed of their testing results. A more detailed one-time, online survey was sent to any participant in March 2021 who had either tested seropositive and/or self-reported having a positive PCR result at any during the study period. Participation was voluntary, and participants could opt out of the study at any time. The protocol (IRB00248772) for this study was approved by The Johns Hopkins Hospital Institutional Review Board.

### 2.2. Survey Questionnaires

Participants were administered a survey at the time of enrollment including demographic, housing, and work practice questions. Following enrollment, participants received weekly surveys via email with structured questions regarding exposures, symptoms, and any PCR testing completed in the previous week.

Any participants who tested seropositive in July or December 2020 and/or reported a positive nasopharyngeal PCR test on their weekly surveys were sent an additional follow-up survey in March 2021 with more detailed questions. The more detailed survey included questions regarding COVID-19 symptoms, care sought for their illness, days missed from work, and perceived long-term effects of SARS-CoV-2 infection (symptoms and/or persisting conditions). All data were entered and reviewed in REDCap.

### 2.3. IgG Sample Testing

At enrollment and at 6 months thereafter, sera were collected from participants to assess the presence of IgG antibodies directed against the S1 subunit of the spike SARS-CoV-2 protein. The assay was performed using a commercial, manual enzyme-linked immunosorbent assay (ELISA) manufactured by Euroimmun (Lubeck, Germany, catalog number EI 2606-9601G) and following modifications of the original protocol, as described in Caturegli et al. [19]. This assay has excellent sensitivity (97%) and specificity (98%). Sera were considered positive for spike antibodies when yielding an optical density ratio >1.23 units.

### 2.4. Analysis

Data were cleaned and analyzed in R [20] and Excel. Logic checks and other quality-control measures were conducted at multiple times throughout the data collection process and at the time of analysis. Demographic characteristics and behaviors of participants with and without antibodies were compared through frequencies, longitudinal patterns, and other descriptive statistics. Comparative statistics for demographic characteristics and behaviors were not used in the analysis, due to the low number of positive cases and relatively small sample size. A chi-square test was used to compare seropositivity and PCR positivity.

## 3. Results

### 3.1. Seropositivity

**Figure 1:**
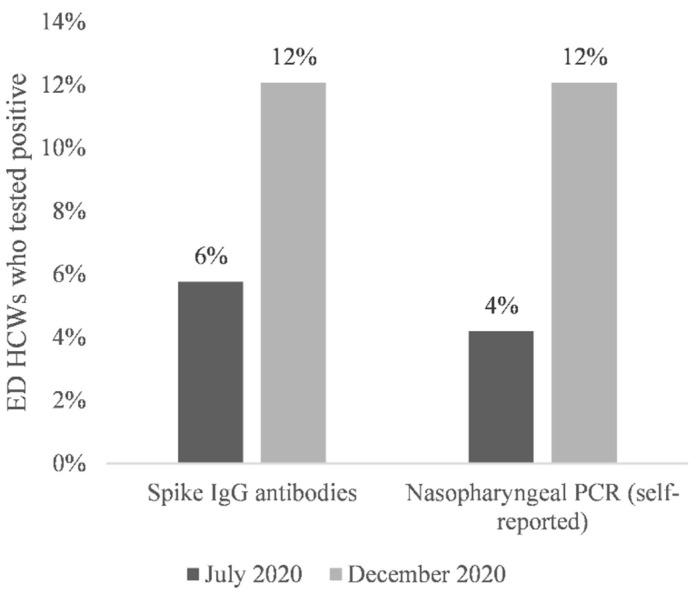
Seropositivity and PCR Positivity.

**Figure 2:**
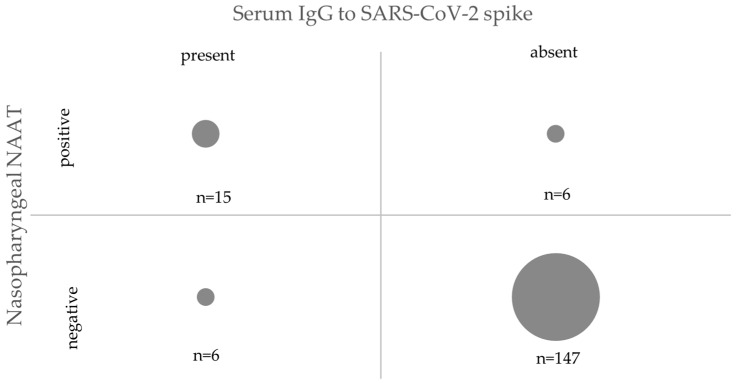
Seropositivity and PCR Positivity Concordance.

At baseline (July 2020), 11 of 191 (5.8%) ED HCWs tested positive for IgG antibodies, and of them, 8 also self-reported a positive nasopharyngeal PCR test (Figure 1). This prevalence increased twofold at the 6-month follow-up (December 2020) when 21 of 174 ED HCWs tested positive for antibodies (Figure 1). All ED HCWs who tested positive in July 2020 remained seropositive in December 2020. Among the 21 seropositive HCWs, 15 (71.4%) reported having had a positive PCR test (8 at baseline and 7 during the 6-month study period), 3 reported having had a negative PCR test result, and 3 reported never having been tested. The frequency of a self-reported PCR test was significantly higher in the seropositive ED HCWs (15 of 21, 71%) than in the seronegative ones (6 of 153, 4%, *p* < 0.001 by chi-squared test, Figure 2). Comparison between antibody and PCR testing showed excellent agreement: 162 of 174 (93%) were either negative (*n* = 147) or positive on both tests (*n* = 15, Figure 2). The remaining 12 discrepancies were either PCR single positive (*n* = 6) or antibody single positive (*n* = 6). Of the six PCR single positives, five reported PCR positivity in November or December 2020.

### 3.2. ED HCWs Characteristics and Seropositivity

Table 1 details demographic, housing, and work practice characteristics of the ED HCWs and associated seropositivity.

#### 3.2.1. Demographics

The age range of the 191 ED HCWs was 20–64 years (mean: 35 years, median: 32). Among the seropositive, 13 of 21 (61.9%) were between the ages of 25 and 34, representing 49% of the total study population. In all, 145 of 191 (75.9%) ED HCWs were female, 45 (23.6%) were male, and 1 (0.5%) was transgender. Seropositivity did not differ between males and females in either July or December 2020.

The majority of the ED HCWs, 117 (61.2%), were White; of the remaining, 41 (21.5%) were African American, 22 (11.5%) were Asian American, 1 (0.5%) was American Indian, and 1 (0.5%) was Native Hawaiian. At baseline, July 2020, the highest seropositivity was found among Asian Americans, 2 of 22 (9.1%) seropositive, followed by 3 of 41 (7.3%) African Americans and 5 of 117 (4.3%) White participants. In December 2020, seropositivity remained highest among Asian Americans at 4 of 20 (20.0%), followed by African Americans with 4 of 33 (12.1%), and White participants with 11 of 112 (9.8%).

#### 3.2.2. Housing

Overall, 132 of 191 (69%) ED HCWs reported living in a multifamily housing unit, 57 of 191 (29%) reported living in single-family homes, and 2 of 191 (2%) reported living in other housing alternatives. Moreover, 17 of 118 (14.4%) ED HCWs living in multifamily units were seropositive in December 2020 versus 4 of 52 (7.4%) living in single-family homes. Similar differences in seropositivity by dwelling type were observed in July 2020. Regarding differences in seropositivity based on household make-up, those living with children under the age of 5 had the highest seropositivity, with 4 of 37 (10.8%) seropositive in July and 5 of 33 (15.2%) seropositive in December.

#### 3.2.3. Work Practices

The highest seropositivity rates were observed among nurses and ED residents. In July, 6 of 84 (7.1%) nurses were seropositive and 4 of 34 (11.8%) residents were seropositive. Nurses and residents accounted for 10 of 11 (90.9%) seropositive cases in July 2020 but constituted only 61.8% of the total study population. By December, 11 of 76 (14.5%) nurses were seropositive and 6 of 31 (19.4%) residents were seropositive. Combined, nurses and residents made up 17 of 21 (80.9%) seropositive cases in December 2020 but only 61.4% of the study population. Furthermore, 2 of 24 (8.3%) patient care technicians and 2 of 16 (12.5%) administration and registration staff were seropositive in December 2020; only one administration staff was positive in July 2020. No attending physicians, advanced patient providers, imaging technicians, or non-clinical staff tested seropositive at either time point. Of those who worked more than 40 h, 17 of 105 (16.2%) were seropositive compared to 4 of 68 (5.9%) who worked fewer than 40 h per week. Seropositivity rates did not differ between those who worked night versus day shifts.

### 3.3. Symptoms and Severity

Over the duration of the study, 11 of 21 (52%) seropositive ED HCWs reported an acute loss of taste and/or smell versus 3 of 153 (2%) seronegative HCWs. In addition, two of the three seronegative HCWs reporting acute loss of taste and/or smell reported a positive PCR test. Comparing weekly reporting from seropositive versus seronegative HCWs, 15 of 21 (71%) seropositive HCWs reported having experienced a cough (versus 52 of 153 (34%) seronegative, 5 of whom were PCR positive/seronegative), 14 (67%) seropositive HCWs reported having had body aches (versus 53 of 153 (35%) seronegative, 5 of whom were PCR positive/seronegative), and 13 (62%) seropositive HCWs reported having fever or fatigue at least once (versus 63 of 153 (33%) seronegative, 6 and 5 of whom were PCR positive/seronegative), respectively (Table 2).

The more detailed follow-up survey administered to subjects with any positive test (serology and/or self-reported PCR) found the following: 18 of the 27 (66.7%) responded, which included 14 of 21 (66.6%) seropositive participants and 4 of 6 (66.7%) self-reported PCR-positive/seronegative participants. Moreover, 12 of 18 (66.7%) ED HCWs (who were seropositive and/or PCR positive) reported having had symptoms that they attributed to their COVID-19 illness, with body aches and fatigue being the most common (10 of 12 (83.3%)), followed by cough (8 of 12 (66.7%)), fever and acute loss of smell/taste (7 of 12 (58.3%)). Among all ED HCWs who reported symptoms, there was a median of 8.5 days of missed work (range: 2–21 days). Of those who had symptoms, 10 of 12 (83%) treated their symptoms with over-the-counter medications and did not seek medical care. Two reported that they had either a telemedicine or outpatient visit with a clinician due to their illness. No participant reported hospitalization, supplemental oxygen usage, or ventilator support for their illness. Furthermore, 6 of 18 (33.3%) ED HCWs stated that they experienced long-term effects from their illness including fatigue (5 of 6), shortness of breath (3 of 6), and cough (2 of 6).

## 4. Discussion

This is the first study, as far as we are aware, examining ED HCW SARS-CoV-2 seropositivity trends over an extended period of time. We observed that HCW seropositivity doubled during the earliest phases of the pandemic, from 5.8% in July 2020 to 12.1% in December 2020. Given that no staff member had received a COVID-19 vaccine during that period, all seroconversions can be attributed to SARS-CoV-2 exposures.

Numerous previous reports have described cross-sectional seropositivity in HCWs during the earliest phases of the pandemic, from early to mid-2020 [1,8,9,10,11,12,13,14,15,16,21]. One systematic review that included studies from the start of the pandemic through August 2020 reported roughly similar overall seropositivity among HCWs across the globe of 8.7% (95% confidence interval 6.7–10.9%) with seropositivity of 12.7% (95% confidence interval 8.6–17.5%) in North America; rates in varied settings ranged from 0% to 45.3% in the sampled studies [7]. Our ED also participated in a multisite study characterizing HCW seropositivity rates across 20 geographically diverse university-affiliated EDs, where an overall seropositivity of 4.6% (95% confidence interval 2.8–7.5%) was described (sampling from 13 May to 8 July 2020) [21]. Those findings are roughly similar to what was observed herein with slight differences attributed to the sampling time frame and regional variability.

Similar to other international and U.S.-based studies that reported relatively low overall disease among infected HCWs [5,6], we observed that although most of our seropositive ED HCWs had symptoms, the vast majority were mild, with very few seeking medical attention. The absence of severe disease may have been due to the relatively younger age of the seropositive participants but could also be related to the relatively low numbers of cases observed. In terms of work disruption, the median number of missed workdays attributed to acute COVID-19 infection was 8.5 days, with all reporting being able to return to work, indicating that COVID-19 illness was not a large contributor to workforce absenteeism, despite early predictions.

Despite mounting evidence that the primary route of SARS-CoV-2 HCW exposure and infection is through the community [8,16], at least up until the recent variant spike associated with the Omicron variant (where data are still emerging), our study found that those individuals with more clinical contact were more likely to seroconvert. In both phases of the serosurvey, the vast majority (90.9% in July (10 of 11), and 80.9% in December (17 of 21)) of the seropositive cases were either nurses or ED residents. Both groups were over-represented relative to their distribution in the population enrolled (nurses and residents combined represented 61.8% of the total study population). This finding may reflect the close and more prolonged interactions that nurses and residents have with patients compared to other clinical and non-clinical staff and is consistent with several other studies that reported higher seropositivity rates among nurses relative to other healthcare workers [5,8,22].

The increasing rates of seroconversion among the ED HCWs observed in this study are likely related in part to increasing community transmission during the 6-month study period. In Baltimore City, MD, USA, where this hospital is located, case counts more than tripled (312%) between July and December 2020 [23]. The largest increase occurred in December 2020, when case counts increased 88% compared to the prior month of November. That increase mirrored national trends with a sharp, steep increase in late winter [24]. In our study, 8 of 13 (61.5%) participants reported a positive PCR result in their weekly survey in November and December 2020, mirroring the local trend seen in Baltimore City. Community transmission may have further increased risk, explaining why those HCWs living with children under the age of five or living in multifamily units had higher seropositivity—through potential opportunities for exposure through childcare arrangements or neighbors.

There are several limitations to this study. First, this study was conducted at a single-site urban tertiary care academic hospital with a relatively robust infrastructure in pandemic preparedness (i.e., HCWs at this hospital had greater access to and training in use of PPE than HCWs at many hospitals in the USA). This may limit comparability to other medical centers. Notably, however, the ED HCW seropositivity rates we observed in July 2020 were similar to those reported in a national ED HCW study [22]. Second, our sample size was small (both with regard to the overall population we followed and the number of reported positive cases), restricting this report to a purely descriptive analysis and preventing us from drawing firmer conclusions regarding the association between demographics and risk of seroconversion. Third, the study was done before the emergence of highly transmissible variants of SARS-CoV-2 such as Alpha, Delta, and now Omicron, so it may underestimate the current potential risk of infection for HCWs both in the occupational and community settings. Nonetheless, the study offers insight regarding changes in seropositivity over an extended 6-month period. Fourth, the reported COVID-19 data from Baltimore City was based on PCR data rather than on serologic data. The fact that some of our ED HCW participants likely resided outside the city proper (where community rates may differ) and that we did not capture community contact history limited our ability to perform direct hospital versus community comparison; still, the collected information allowed some broad inferences about temporal trends. Lastly, we did not capture data regarding the number of missed workdays for seronegative ED HCWs during this period; accordingly, we cannot comment on comparative impact of COVID-19-related missed workdays versus missed workdays for other reasons.

In summary, the SARS-CoV-2 seropositivity rate of ED HCWs doubled between July and December 2020 at this urban tertiary care academic medical center, an increase that was relatively less than that observed in the surrounding community during the same time period. The severity of reported illness was low among seropositive ED HCWs. Thus, at this particular hospital ED, concerns regarding high rates of disease and associated ED HCW workforce shortages were not borne out, at least during the earliest phases of the pandemic. While reassuring, at least on the local level, our findings may have been impacted by the fact that HCWs at this site had consistent access to PPE and training on proper usage. There may have been wide variation in PPE availability and training among other hospitals in the USA [25]. The extensive spread of new variants (Alpha, Delta, and Omicron) once again places HCW exposure, transmissions, and associated workforce shortages at the forefront. Given different transmission dynamics associated with emerging variants, ongoing surveillance and research is needed to help mitigate individual risk and optimize the health and well-being of staff at the front lines. The ability of variants such as Delta and Omicron to evade vaccine-induced immunity further underscores the continued need to assess the impact of COVID-19 on HCWs. This is not only important for preserving the safety and health of HCWs themselves but also critical to keep EDs (and other components of the hospital) functional and able to deliver care to the communities they serve when healthcare systems are stressed.

## Figures and Tables

**Table 1:** Seropositivity of SARS CoV-2 and related characteristics.

	July 2020			December 2020		
Characteristics	%	Positive	Total	%	Positive	Total
Total	5.8%	11	191	12.1%	21	174
Age Group						
20−24	0.0%	0	18	6.3%	1	16
25−29	5.9%	3	51	12.5%	6	48
30−34	6.8%	3	44	18.4%	7	38
35−39	6.1%	2	33	6.9%	2	29
40−44	16.7%	2	12	16.7%	2	12
45−49	0.0%	0	13	9.1%	1	11
50−54	0.0%	0	6	16.7%	1	6
55−59	11.1%	1	9	11.1%	1	9
60−64	0.0%	0	5	0.0%	0	5
Sex						
M	6.7%	3	45	12.2%	5	41
F	5.5%	8	145	12.1%	16	132
Transgender Male	0.0%	0	1	0.0%	0	1
Race						
White	4.3%	5	117	9.8%	11	112
Black or African American	7.3%	3	41	12.1%	4	33
Asian	9.1%	2	22	20.0%	4	20
American Indian or Alaska Native	0.0%	0	1	0.0%	0	1
Native Hawaiian or Other Pacific Islander	0.0%	0	1	0.0%	0	1
Other	12.5%	1	8	33.3%	2	6
Co-morbidities						
Present	7.3%	4	55	12.2%	6	49
Not Present	5.1%	7	136	12.0%	15	125
Smoking						
Current Smoker	12.5%	1	8	12.5%	1	8
Former Smoker	4.3%	1	23	15.8%	3	19
Never Smoked	5.7%	9	159	11.6%	17	146
BMI						
Underweight	0.0%	0	5	25.0%	1	4
Normal Weight	6.8%	4	59	14.5%	8	55
Overweight	5.1%	3	59	11.1%	5	45
Obese	7.5%	4	53	10.7%	6	56
Pregnant						
Yes	0.0%	0	4	0.0%	0	4
No	5.7%	8	140	12.6%	16	127
Housing						
Multi-unit	7.6%	10	132	14.4%	17	118
Single Family	1.8%	1	57	7.4%	4	54
Other	0.0%	0	2	0.0%	0	2
People in Household						
Kids under 18						
Yes	7.1%	5	70	12.5%	8	64
No	5.0%	6	120	11.9%	13	109
Kids under 5						
Yes	10.8%	4	37	15.2%	5	33
No	4.6%	7	153	11.4%	16	140
Kids in Daycare						
Yes	0.0%	0	4	0.0%	0	3
No	12.1%	4	33	16.7%	5	30
Relatives over 65						
Yes	7.1%	1	14	8.3%	1	12
No	5.7%	10	176	12.4%	20	161
Relatives under 65						
Yes	6.2%	9	145	12.9%	17	132
No	4.8%	2	42	10.5%	4	38
Number of Other People in Household						
0	3.6%	1	28	8.0%	2	25
1	7.7%	5	65	18.3%	11	60
2+	5.1%	5	98	9.0%	8	89
Occupation						
Nurse	7.1%	6	84	14.5%	11	76
Resident	11.8%	4	34	19.4%	6	31
Technician	0.0%	0	28	8.3%	2	24
Registrar	0.0%	0	12	8.3%	1	12
Attending Physician	0.0%	0	10	0.0%	0	10
APP	0.0%	0	7	0.0%	0	7
Imaging Technician	0.0%	0	7	0.0%	0	6
Non-clinical Staff	0.0%	0	5	0.0%	0	4
Administrative	25.0%	1	4	25.0%	1	4
Work Nights						
Yes	2.4%	1	42	11.1%	4	36
No	6.8%	10	147	12.5%	17	136
Average Hours per Week						
<40	1.3%	1	76	5.9%	4	68
≥40	8.8%	10	114	16.2%	17	105

**Table 2:** Symptoms of seropositive and seronegative participants reported in weekly survey.

	Seropositive Participants (*n* = 21)		Seronegative Participants (*n* = 170)	
Symptoms	At Least 1 Report	%	At Least 1 Report	%
Cough	15	71%	52	34%
Body aches	14	67%	53	35%
Fever	13	62%	30	20%
Fatigue	13	62%	63	41%
Sore throat	12	57%	63	41%
Diarrhea	11	52%	45	29%
Acute loss of taste and or smell	11	52%	3	2%
Shortness of breath	8	38%	20	13%
Chills	8	38%	26	17%
Nausea, vomiting	6	29%	25	16%
Chest pain	4	19%	14	9%
Difficulty breathing	2	10%	4	3%
Wheezing	1	5%	3	2%
Repeated shaking with chills	1	5%	7	5%

## Data Availability

The data presented in this study are available on request from the corresponding author. The data are not publicly available due to patient confidentiality.
